# Bone tissue engineering supported by bioprinted cell constructs with endothelial cell spheroids

**DOI:** 10.7150/thno.74852

**Published:** 2022-07-11

**Authors:** WonJin Kim, Chul Ho Jang, GeunHyung Kim

**Affiliations:** 1 Department of Biomechatronic Engineering, College of Biotechnology and Bioengineering, Sungkyunkwan University (SKKU), Suwon 16419, South Korea.; 2 Department of Otolaryngology, Chonnam National University Medical School, Gwangju 61469, South Korea.; 3 Biomedical Institute for Convergence at SKKU (BICS), Sungkyunkwan University, Suwon 16419, South Korea.

**Keywords:** Tissue engineering, bone, bioprinting, spheroids, multiple cells

## Abstract

In bone tissue engineering, efficient formation of vascularized bone tissue is a challenging issue. Here, we introduce a new strategy for effectively using multiple cells laden in a hybrid structure, such as endothelial cell (EC) spheroids and homogeneously distributed human adipose stem cells (hASCs) for bone regeneration.

**Methods:** To fabricate the EC spheroids, cell-mixed mineral oil was used, and microscale droplets of the cell mixture were interlayered between the bioprinted hASC-laden struts. *In vitro* cellular responses of spheroid-laden multiple-cell constructs have been evaluated by comparing with the cell constructs bioprinted with the mixture of hASCs and ECs. In addition, mastoid obliterated rat model has been used to observe *in vivo* bone formation of those cell constructs.

**Results:** The spheroid-laden multiple-cell constructs induced outstanding angiogenesis and osteogenic activities compared to a conventionally bioprinted multiple-cell construct. The enhanced biological results were clearly due to the EC spheroids, which triggered highly cooperative crosstalk between ECs and stem cells. The co-culture of the hASC constructs with the EC spheroids exhibited enhanced osteogenic- and angiogenic-related gene expression *in vitro*. In addition, in a rat obliterated mastoid model, considerably greater new bone formation and more competent development of new blood vessels were observed compared to those achieved with the normally bioprinted multiple cell-loaded structure.

**Conclusion:** *In vitro* and *in vivo* results demonstrated that the bioprinted spheroid-laden multiple-cell construct is a potential candidate for use in bone tissue engineering.

## Introduction

Bone substitutes, such as autografts, allografts, and xenografts, are considered favorable for the repair of bone defects 1-3. In particular, cellular and material sources from substitutes enhance osteogenesis and vascularization of bone defects. However, these substitutes have some problems, including limited sources of donor tissues, possibility of multiple surgical procedures, immune rejection, and potential infection 4-7. For these reasons, tissue engineering has been considered a potential alternative for regenerating bone tissues 8. However, although this method has provided outstanding improvement in the successful regeneration of bone tissues, the lack of appropriate blood vessels remains an issue, because the high degree of vascularization can be regarded as an extremely important process in both bone formation and remodeling 9, 10.

To overcome this shortcoming, two typical methods have been applied to induce the formation of blood vessels in tissue-engineering therapeutic materials. The first is based on three-dimensional (3D) scaffolds combined with angiogenic growth factors, cytokines, and peptides 10-12. In the scaffold-based approach, various bioactive materials and designs of the pore geometries of constructs are used to achieve successful angiogenesis and vascularization 12-14. In general, a high porosity over 70% and interconnected pores (pore diameter over 150 μm) have been considered suitable for inducing vascularization and osteoconductivity as they support the high permeability of nutrients and removal of metabolic waste 15, 16. The second is cell-based approaches with or without biofabrication tools. In these approaches, bone formation is achieved by vasculogenesis and osteogenesis, which are induced by well-known signaling pathways in osteogenic and endothelial cells (ECs) that are activated by signaling molecules secreted in the extracellular region by cell-cell interactions 17-19. Recently, bioprinting methods using osteogenic and angiogenic lineage cells have been used to fabricate vessel networks 20-23. As this technique has enabled precise control of the spatial distribution of cells within cell constructs and multiple-cell printing of endothelial and osteogenic cells, the fabrication of biomimetic artificial bone tissues with efficient vascularization is possible. Bioprinted multiple cell-laden constructs for vascularized bone tissue regeneration have been investigated by various researchers in two distinct ways: vascular patterning 24, 25 and multiple cell printing 20, 26. Tissue-engineered bone with a vascular cell-laden structure exhibited rapid vessel formation in the construct within 7 days of cultivation 24, and multiple-cell-laden constructs using stem cells and ECs showed significantly higher osteogenesis than stem cell constructs 25. However, the limited survival of embedded cells in a volumetric cell construct can still be a problem in the fabrication of vessel networks using biofabrication methods.

Here, we suggest a new bioprinted cell construct consisting of EC spheroids, which can be widely used in tissue regeneration owing to its *in vivo*-like 3D microenvironment to induce efficient cell-to-cell interactions through various signaling factors 27, 28 and homogeneously distributed human adipose stem cells (hASCs) supported by two biocompatible materials, decellularized extracellular matrix (dECM) derived from porcine bone tissue and β-tricalcium phosphate (β-TCP), which are outstanding bioactive materials that induce efficient bone formation. The EC spheroids laden in the hASC constructs were fabricated simultaneously for bioprinting. By positioning droplets of a mixture of mineral oil and ECs, the cell spheroids were well interlayered between the bioprinted struts containing hASCs. The spheroid-laden multiple-cell constructs induced outstanding angiogenesis and osteogenic activities compared to a normally bioprinted dECM/β-TCP multiple-cell construct, as demonstrated by the immunochemistry and expression of several genes related to angiogenesis and osteogenesis. The enhanced biological results were clearly due to the EC spheroids, which induced highly cooperative crosstalk between ECs and stem cells. Furthermore, when the bioprinted cell constructs were applied in a rat obliterated mastoid model, as expected, considerably greater new bone formation and more competent development of new blood vessels were observed compared to those achieved with the normally bioprinted multiple cell-loaded structure.

## Materials and methods

### Cells and bioinks

In this study, human adipose stem cells (hASCs) (Lonza, USA) and human umbilical vein endothelial cells (HUVECs; Lonza) were used. The cells were cultured at 37 °C and 5% CO_2_ with different culture media, namely, growth medium (GM) for hASCs consisting of Dulbecco's Modified Eagle's Medium-low-glucose (DMEM-L; Sigma-Aldrich, USA), 10% fetal bovine serum (FBS; BioWest, USA), and 1% penicillin-streptomycin (PS; Thermo-Fisher Scientific, USA) and EBM^TM^-2 (Lonza) for HUVECs supplemented with the EGM^TM^-2 endothelial SingleQuots^TM^ kit (Lonza) and 1% PS (EBM). The culture medium was changed every 2 days.

Before formulating the stem cell-loaded bioink, a porcine bone-derived dECM (BdECM) sponge, prepared using a previously described demineralization/decellularization protocol (described in Supplementary materials) 29, was dissolved in deionized water and neutralized by mixing with 10×DMEM (Sigma-Aldrich) at a ratio of 1:1. The BdECM hydrogel was then mixed with b-TCP and hASCs (1.2 × 10^7^ cells/mL). The final concentrations of dECM and- TCP were 50 and 200 mg/mL, respectively. To obtain cell spheroids, mineral oil (Sigma-Aldrich) containing HUVECs (2.0 × 10^7^ cells/mL) was used. The total cell density to fabricate the cell-constructs was fixed as 2.0 × 10^7^ cells/mL in which the ratio of the hASCs and ECs was 3:2.

### Formation of cell spheroids in the BdECM hydrogel

HUVEC-loaded mineral oil and BdECM hydrogel (5 wt %) were used to observe the formation of EC spheroids in the mineral oil. In the BdECM hydrogel loaded in 48-well plates, mineral oil droplets (~0.05 µL) containing various densities of HUVECs (0.5-3.0 × 10^7^ cells/mL) were placed in the hydrogel using a micropipette (Gilson Inc., France). GM was used to culture the loaded cells and was changed every 2 days.

### Preparation of conventional EC spheroids using an agarose micro-well

Conventional EC spheroids (~200 µm in diameter) were prepared using a nonadherent agarose mold according to the manufacturer's protocols 30. Agarose (2%; Invitrogen), solved in Dulbecco's phosphate buffered saline (DPBS; Biowest), was used to obtain an agarose mold. HUVECs (2.56 × 10^5^ cells per mold) were then cultured in an agarose mold with GM for 3 days.

### Enzyme-linked immunosorbent assay (ELISA)

To observe the growth factor secretion ability of the EC spheroids prepared with the mineral oil droplets, ELISA assays for human vascular endothelial growth factor (VEGF; Invitrogen) and bone morphogenic protein 2 (BMP-2; Antigenix America Inc., USA) were performed according to the manufacturer's protocols. Monocultured HUVEC and EC spheroids prepared with the conventional microwell and mineral oil were treated with serum-free DMEM-L (1 mL) for 1 day at 37 °C under a CO_2_ environment. VEGF and BMP-2 levels in the conditioned media were measured using ELISA plates and a microplate reader at 450 nm. Known standards were used to determine the VEGF and BMP-2 concentrations (n = 3).

### Bioprinting process to obtain cell constructs

A 3D bioprinting system (DTR3-2210 T-SG; DASA Robot, South Korea) consisting of an AD3000C dispenser (Ugin-tech, South Korea) and a dual-barrel equipped with two nozzles (hASC/BdECM/β-TCP bioink:25G nozzle and HUVEC-loaded mineral oil:30G nozzle) was used at a controlled temperature (barrel/nozzle: 25 °C and printing plate: 37 °C). The printing speed (5 mm/s) and pneumatic pressure (90 kPa) were used to obtain the hASCs/BdECM/b-TCP struts. In addition, HUVEC-loaded mineral oil droplets were placed on the two struts at a printing speed (5 mm/s) and an oil flow rate (3.8 µL/min).

As a control cell construct, HUVECs (0.8 × 10^7^ cells/mL) and hASCs (1.2 × 10^7^ cells/mL) were mixed with the BdECM (50 mg/mL)/β-TCP (200 mg/mL) solution, and the bioink was printed using the same printing conditions. The control cells were cultured in GM at 37 °C in a 5% CO_2_ environment. The GM was changed every 2 days.

### Characterization of the cell constructs

The surface and pore geometry of the fabricated cell structures were characterized using an optical microscope and SNE-3000M scanning electron microscope (SEM) (SEC Inc., South Korea). Before visualizing the surface morphology of the biocomposites, the structures were fixed, dehydrated using 10% neutral buffered formalin (NBF; Sigma-Aldrich) and an ethanol series (50%, 60%, 70%, 80%, 90%, and 100%), and freeze-dried. The morphological structure was measured using the ImageJ software (National Institutes of Health, USA). All values are reported as mean ± standard deviation (SD).

### *In vitro* cellular responses

The Cell Proliferation Kit I (Boehringer Mannheim, Germany) was used to perform the 3-(4,5-dimethylthiazol-2-yl)-2, 5-diphenyltetrazolium bromide (MTT) assay. The samples were rinsed three times with DPBS and treated with the MTT solution for 4 h at 37 °C to induce the production of purple formazan crystals from metabolically active cells. Then, a sodium dodecyl sulfate-containing solubilization solution was added to dissolve the crystals. A microplate reader (Epoch^TM^; BioTek, South Korea) was used to measure the optical density (OD) of the colored solution at 570 nm. All values are reported as mean ± SD (n = 4 or 7).

To observe cell viability, cells were stained with calcein AM (0.15 mM; Invitrogen) and ethidium homodimer-1 (2 mM; Invitrogen). The LSM 700 confocal microscope (Carl Zeiss, Germany) was used to visualize the stained cells. Cell viability was estimated by counting the number of live (green) and dead (dead) cells using the ImageJ software. All values are reported as mean ± SD (n = 4).

To observe the distribution of the hASCs and EC spheroids in the printed cell structure, Cell-Tracker^TM^ (Molecular Probes, USA) was used to pre-stain the cells according to the manufacturer's protocol. The cultured hASCs and HUVECs were harvested, followed by incubation in a staining solution (37 °C) for 30 min before formulating the bioinks. After printing the bioinks, hASCs (red) and EC spheroids (green) were visualized using a confocal microscope after 3 days of culture.

The morphology of the cultured cells and formed EC spheroids was observed by staining nuclei and F-actin with diamidino-2-phenylindole (DAPI) (Invitrogen) and Alexa Fluor 594-conjugated phalloidin (Invitrogen), respectively. Briefly, cells were fixed with 10% NBF for 30 min and permeabilized with 0.1% Triton X-100 (Sigma-Aldrich) for 10 min at 37 °C. The treated cells were then treated with a staining solution containing DAPI (1:100 in DPBS) and phalloidin (1:100 in DPBS) for 1 h at 37 °C. The stained cells were observed under a confocal microscope to visualize the nuclei (blue) and F-actin (red).

In addition, the diameter (n = 17) and area (n = 10) of the cell spheroids, length (n = 20) of the capillary sprouts determined with F-actin, and number of connected sprouts (n = 3) were quantified using the ImageJ software. All values are reported as the mean ± SD.

### Immunofluorescence

To evaluate the differentiation of hASCs and ECs, the cells were treated with NBF (10%; fixation) for 1 h, bovine serum albumin (BSA; Sigma-Aldrich) (2%; blocking) for 2 h, and Triton X-100 (2%; permeabilization) for 2 h. The cells were then treated with an anti-mouse Ve-cadherin primary antibody (5 μg/mL; Invitrogen), an anti-rabbit CD31 primary antibody (5 μg/mL; Invitrogen), and an anti-mouse osteopontin (OPN) primary antibody (5 μg/mL; Invitrogen) overnight at 4 °C, followed by staining with the Alexa Fluor 488-conjugated anti-mouse secondary antibody (1:50 in DPBS; Invitrogen) and Alexa Fluor 594-conjugated anti-rabbit secondary antibody (1:50 in DPBS; Invitrogen) for 1 h according to the host species of the primary antibody. The cells were counterstained with DAPI (5 μM in DPBS) to visualize nuclei. The stained cells were observed under a confocal microscope. Quantification of the OPN^+^ and CD31^+^ areas was performed using ImageJ software. All values are reported as mean ± SD (n = 4).

### Quantitative reverse transcription polymerase chain reaction (RT-qPCR)

Gene expression was evaluated by RT-qPCR, using the 2^-∆∆CT^ method. Total RNA was isolated by treating the cultured cells with TRIzol reagent (Sigma-Aldrich). An FLX800T spectrophotometer (Biotek, USA) was used to measure the purity of the isolated RNA. cDNA was synthesized from RNase-free DNase-treated RNA by reverse transcription using the ReverTraAce™ qPCR RT Master Mix (Toyobo Co., Ltd., Japan). To perform RT-qPCR analysis, threshold cycle (CT) values were measured using the synthesized cDNA and Thunderbird^®^ SYBER^®^ qPCR mix (Toyobo Co., Ltd.) *via* a StepOnePlus PCR system (Applied Biosystems, USA). The expressed gene levels were normalized using the measured values of the housekeeping gene glyceraldehyde 3-phosphate dehydrogenase (*Gapdh*). All values are reported as mean ± SD (n = 3 or 4). The details of the primers used are listed in Table S1.

### Surgical procedure for bulla obliteration

All animal experiments were approved by the Institutional Animal Care and Use Committee of Chosun University (CIACUC2020-A0017). Sprague-Dawley rats (n = 12, male, weighing 200-250 g) with a healthy eardrum and Preyer's reflex were purchased from Samtakobio (South Korea). After shaving the periauricular hair using an electric clipper, antiseptic paintings using povidone-iodine solution were performed on the periauricular region. An initial incision was made in the periauricular skin, covering an appropriate region of the bullae under general anesthesia using isoflurane. To reduce local pain and bleeding during skin incisions, dental lidocaine (1:100,000) was topically administered around the periauricular region. The right bulla was exposed by subcutaneous dissection using mosquito forceps, and blood was coagulated using a portable cordless Bovie® pocket cautery (pocket cautery; Pioneer Co., UK). The bony wall of the bulla cavity was opened by drilling and irrigated with PBS to remove the remaining dust from the bulla cavity. The bullae were filled with the control (n = 6) and experimental (n = 6) constructs. After mastoid obliteration, the round bony hole was covered with a compressed spongy sheet (Johnson & Johnson Co., NJ, USA). The wounds were then sutured using an auto-clipper. Intramuscular injection using an antibiotic (ciprofloxacin) was performed.

### Fluorescent labeling and multiphoton microscopy

After 8 weeks of healing, the dynamics of the newly formed bones were observed by injecting them with fluorescent bone marker labels. At 2 weeks, alizarin red S (20 mg/kg body weight), oxytetracycline HCl (20 mg/kg body weight), and xylenol (20 mg/kg body weight) (Sigma-Aldrich) were administered *via* intraperitoneal injection. All dyes were prepared with saline immediately before use. To visualize remodeled bone, an intravital multiphoton microscope (SP8-MP; Leica, Wetzlar, Germany) with a water immersion lens (25×, 0.9 NA) was used. The fluorophores were excited using the InSight DS Plus laser system (Spectra-Physics, Santa Clara, USA) at the following wavelengths: xylenol (excitation = 800 nm, emission = 375-570 nm), oxytetracycline (excitation = 1040 nm, emission = 365-490 nm), and alizarin (excitation = 1180 nm, emission = 538-580 nm). Alizarin -, oxytetracycline-, and xylenol-positive areas were quantified using the ImageJ software. All values are expressed as mean ± SD (n = 4).

### Bone histomorphometric analysis with micro-computerized tomography (CT)

To observe the formation of regenerated bone, *ex-vivo* micro-CT (SkyScan 1173; Bruker MicroCT N.V., Belgium) was performed. The rats were sacrificed 8 weeks after transplantation. Following fixation of the dissected specimens with 10% formalin *via* eardrum perforation, micro-CT was performed on the fixed samples using a Quantum GX μCT imaging system (PerkinElmer, USA) at KBSI. The X-ray parameters were as described in our previous study 31; the X-ray source was set to 90 kV/80 μA. Furthermore, a 45 mm view field, 90 μm voxel size, high resolution scan mode, 4 min scan time, and 162 mGy per scan approximate X-radiation dose were utilized.

### Histological examination

After micro-CT examination, the bulla tissues were decalcified in 10% ethylenediaminetetraacetic acid (EDTA) (National Diagnostics, USA), dehydrated with ethanol and xylene, and embedded in paraffin. The paraffin blocks were sectioned at 5 μm thickness using a microtome (LEICA RM2255; Leica). After deparaffinization, histological, immunohistological, and immunochemical staining were performed. For histological analysis, hematoxylin and eosin (H&E) staining and Mason's trichome staining (MTS) assay were performed according to standard protocols 31. The newly formed bone and mature bone areas were estimated using the ImageJ software. All values are expressed as mean ± SD (n = 4).

Tissue sections were immunohistologically analyzed by staining for 24 h with an osteocalcin (OCN) antibody (1:3200; Santa Cruz, USA), Discovery UltraMap anti-mouse horseradish peroxidase (HRP), and UltraMap anti-rabbit HRP (Roche Diagnostics Ltd., Switzerland). The stained sections were observed using a slide scanner (Pannoramic 250 Flash III; 3DHistech, Hungary). OCN-positive areas were calculated using the ImageJ software. All values are expressed as the mean ± SD (n = 4).

Immunofluorescence staining of vessel formation was performed using anti-CD31 (5 μg/mL; Invitrogen) primary antibody and Alexa 594-conjugated secondary antibody (1:200; Invitrogen). The specimens were counterstained with DAPI (1:500; Sigma-Aldrich) for 10 min. The stained slides were visualized using a Zeiss confocal microscope. CD31-positive areas were quantified using the ImageJ software. All values are expressed as mean ± SD (n = 4).

### Statistical analysis

A student's *t*-test was used (two groups), and a single-factor analysis of variance (ANOVA) with Tukey's honest significant difference (HSD) post-hoc test (three or more groups) was performed to evaluate the statistical analyses using the SPSS software (SPSS, Inc., USA). Values of ^*^p < 0.05, ^**^p < 0.01, and ^***^p < 0.001 were considered statistically significant.

## Results and Discussion

### Preparation of EC spheroids fabricated using mineral oil

It is widely known that the 3D cellular environment is preferred to the two-dimensional (2D) cellular environment because the 3D environment is very similar to an *in vivo*-like microenvironment, inducing realistic spatial cell-cell or cell-substrate interactions 32. Because of their geometrical and biological resemblance to native tissues, cell spheroids have been widely applied in various tissue engineering applications 27, 33. However, despite their outstanding performances, such as high levels of tissue-specific gene expression, compared to that of 2D culture systems, the time-intensive spheroid fabrication and complicated procedures that integrate other biofabrication methods (such as bioprinting and electrospinning) to obtain complex 3D cell constructs have limited their therapeutic application in tissue regeneration.

**Figure 1:**
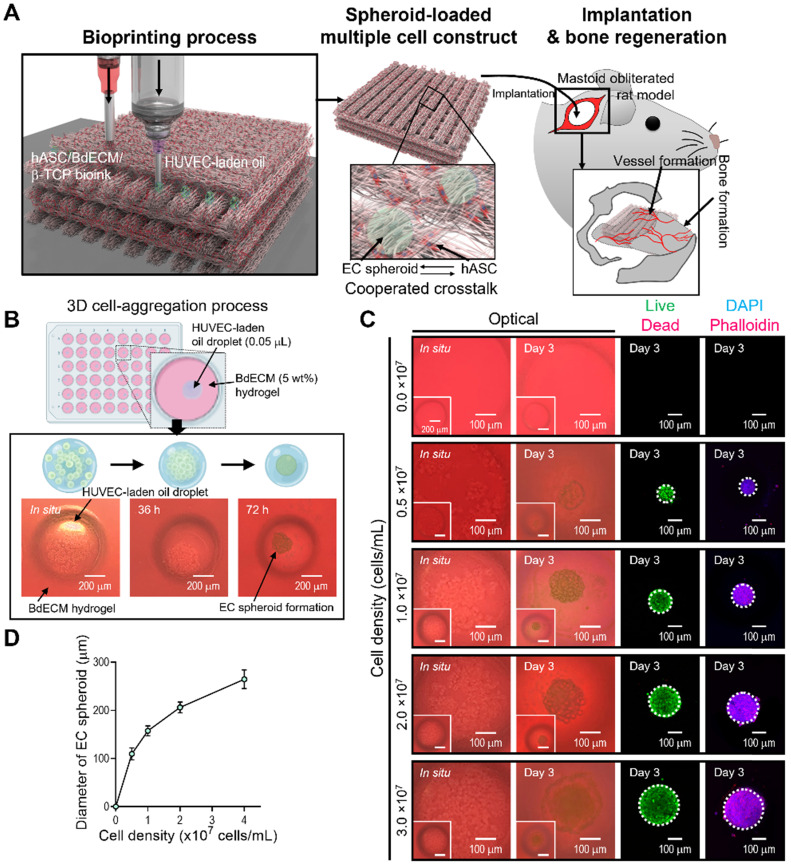
**EC spheroid formation using cell-laden mineral oil droplets. (A)** Schematic diagram exhibiting the fabrication of EC spheroid-loaded multiple-cell construct and *in vivo* evaluation. **(B)** Schematics describing the principle of cell spheroid formation and optical images of endothelial cell (EC) spheroids using an EC-laden mineral oil droplet and a bone-derived decellularized extracellular matrix (BdECM) hydrogel (5 wt%). **(C)** Optical, live (green)/dead (red), and dapi (blue)/phalloidin (red) images showing EC spheroid formation for various cell-densities (0.0, 0.5, 1.0, 2.0, and 3.0 × 10^7^ cells/mL) laden in mineral oil. **(D)** Diameter of self-assembled EC spheroids for the cell densities (n = 17).

Here, we propose a new biofabrication method to obtain a hybrid cell structure, in which bioprinted struts and cell spheroids are easily combined to regenerate vascularized bone tissue (Figure 1A). To achieve this goal, a method for fabricating cell spheroids that can be instantaneously and simply combined with a conventional bioprinting process is required. To obtain cell spheroids, we developed a spontaneous cell aggregation process in which a drop of a mixture of ECs in mineral oil is released into a hydrogel or confined structure. As shown in Figure 1B, as the cell-laden mineral oil was embedded in the hydrogel, the 3D cell aggregation process could be stably formed owing to the hydrostatic pressure (or external forces) of the hydrogel.

The morphological shape of the spheroids formed using ECs and mineral oil droplets is shown in Fig. 1A. In the initial state of the cell-laden oil droplet, the cells were distributed in a droplet-like monolayer. However, after 72 h, the cells were fully aggregated and formed spheroids. The results revealed that the cells within the mineral oil droplet migrated during the culturing period to form a firm spherical shape. In addition, the spherical morphology was sustained for more than 72 h, as shown in Figure 1C. Therefore, we determined that a fabrication process using mineral oil droplet could be used to successfully obtain cell spheroids within 72 h of cell culture, similar to the hanging drop technique 34.

Live/dead and DAPI/phalloidin images of aggregated ECs are shown in Figure 1C. After 3 days, the cells were fully aggregated, and by manipulating the cells (0-3 × 10^7^ cells/mL) that were mixed with the mineral oil, various sizes of cell spheroids were easily formed (Figure 1D). The aggregated ECs were alive and firmly preserved. The results indicate that the fabrication process using an oil droplet can be an effective and versatile cell spheroid fabrication method that can also be instantaneously combined with a bioprinting process.

### Characterization of EC-spheroids and vascularization ability

**Figure 2:**
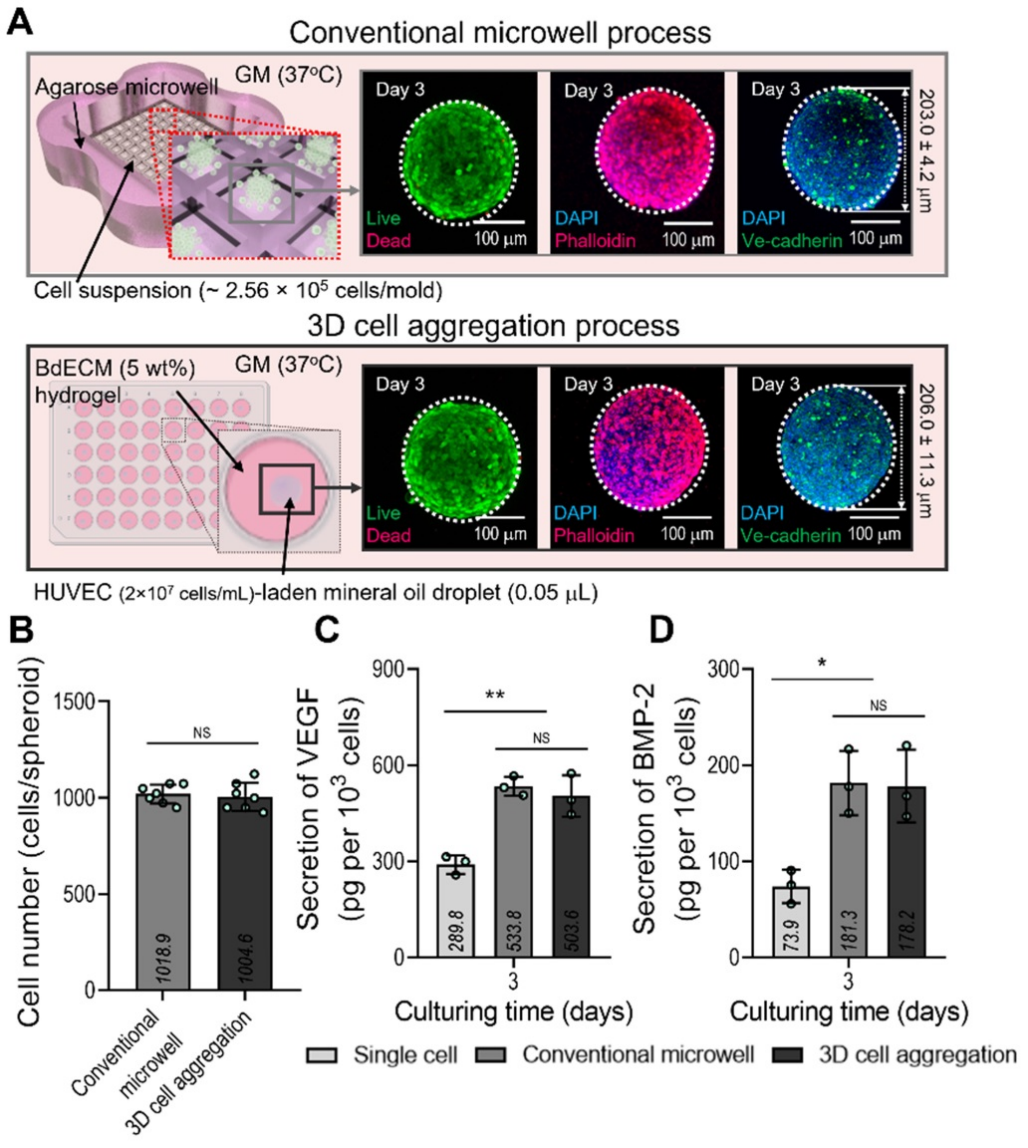
**Comparison of the conventional EC spheroid and spheroid fabricated using the mineral oil. (A)** Schematics showing the spheroid fabrication methods and fabricated EC spheroids characterized with live/dead, dapi/phalloidin, and dapi/Ve-cadherin (green) images at 3 days. **(B)** Number of cells contained in the EC spheroids estimated using the MTT assay. Comparisons of **(C)** vascular endothelial growth factor (VEGF) and **(D)** bone morphogenic protein 2 (BMP-2) secreted from the EC spheroids and single cell two-dimensional culture at 3 days (n = 3). ^*^p < 0.05, ^**^p < 0.01, one-way ANOVA with Tukey's HSD post-hoc test.

We compared the biological factors (VEGF and BMP-2) secreted from the cell spheroids formed using a conventional microwell process (average diameter = 203.0 ± 4.2 µm) and the process using mineral oil droplets (average diameter = 206.0 ± 11.3 µm). ECs and a similar cell density (~1.0 × 10^3^ cells/spheroid) were used in both cases. Figure 2A shows the fluorescence images of live (green)/dead (red), DAPI(blue)/F-actin (red), and DAPI/Ve-cadherin (green) cells cultured for 3 days. As seen in the images, the cells were alive and similar cell-morphological structures of F-actin and positive expression of Ve-cadherin were observed in both cell spheroids consisting of similar cell number (Figure 2B). In addition, the secretion of the two growth factors in the cell spheroids was compared with that in single cells of the same cell density (Figure 2C-D). As expected, VEGF and BMP-2 from both spheroids, which induce osteogenesis and angiogenesis of stem cells, exhibited similar secretion levels. Furthermore, the 2D culture of the single cells showed significantly lower levels of the genes than the two types of cell spheroids.

### Fabrication of a hybrid scaffold laden with EC spheroids and hASCs

**Figure 3:**
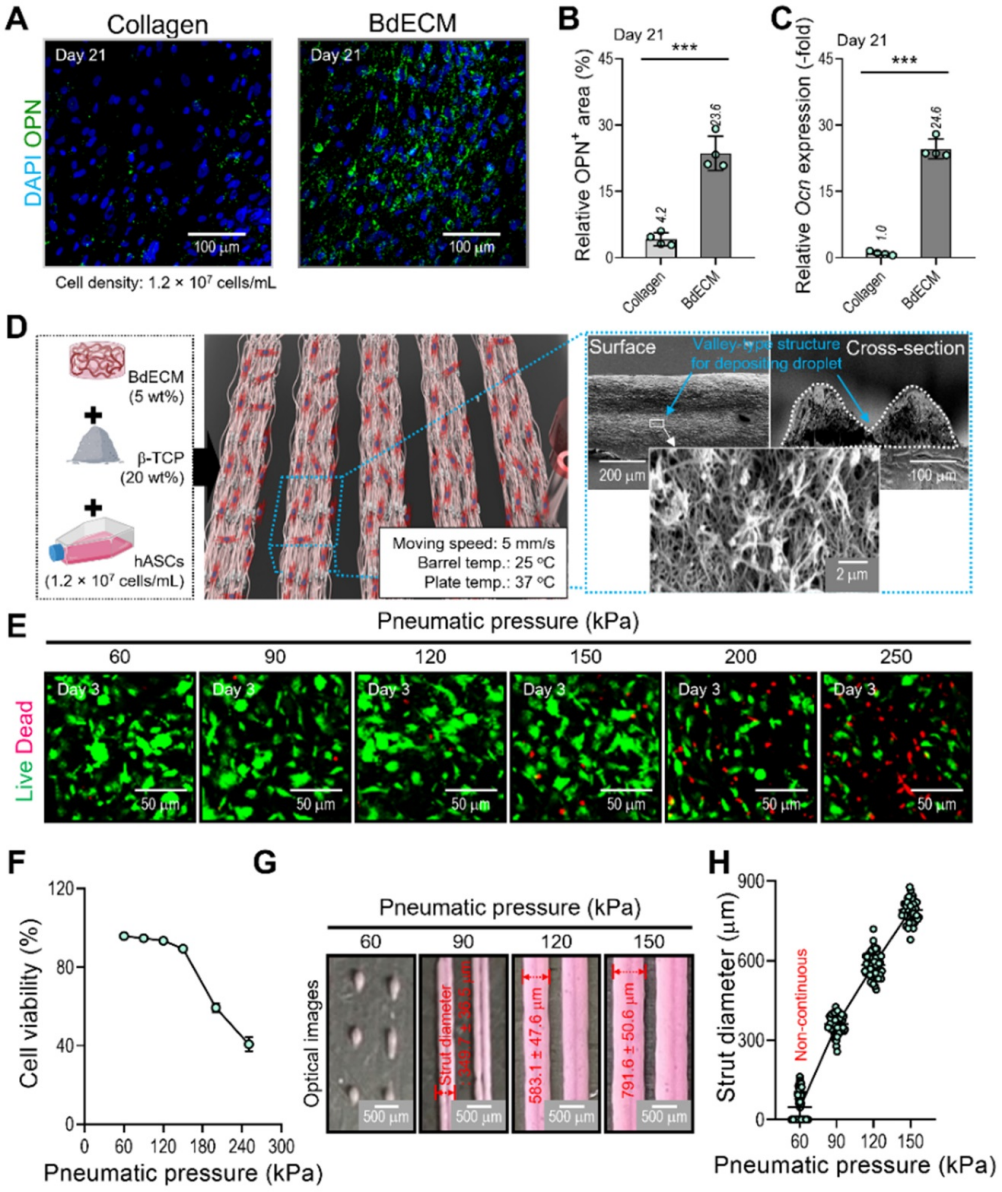
** Characterization of the BdECM bioink and fabrication of hASC-laden BdECM/β-TCP struts.** Comparison of hASC-laden collagen and BdECM bioinks cultured on day 21: **(A)** DAPI/osteopontin (OPN; green), **(B)** OPN^+^ area using the OPN images (n = 4), and **(C)** relative *Ocn* expression level (n = 4). **(D)** A schematic showing the bioink formulation and bioprinted two-parallel hASC-laden BdECM/β-TCP struts. **(E)** Live/dead images and **(F)** cell-viability (n = 4) at 3 days of the bioprinted hASC/BdECM/β-TCP struts for various pneumatic pressures (60-250 kPa). **(G)** Optical images showing the printed struts for various pneumatic pressures and **(H)** measured diameter of the two-parallelly printed struts (n = 50). ^***^p < 0.001, Student's *t*-test.

It is well known that the BdECM derived from porcine bone tissue can induce osteogenic activities of encapsulated hASCs much better than the pure collagen bioink. We also evaluated the osteogenic activity of collagen and BdECM bioinks laden with hASCs. Figure 3A-C shows the immunofluorescence image (DAPI/OPN), OPN area (%), and the OCN expression for the BdECM and collagen bioink at 21 days. As expected, the results indicated that the BdECM bioink can be much more helpful for the osteogenic activities of hASCs than the pure collagen bioink. The detailed constituents of BdECM are described in the supplemental information (Figure S1A-C).

We used the BdECM (50 mg/mL)/β-TCP (200 mg/mL)/hASCs (1.2 × 10^7^ cells/mL) bioink to fabricate cell-laden struts, which were used in the fabrication of EC spheroids. The β-TCP composition of the bioink was selected based on our previous study 35, 36, and the mixture ratio was the most appropriate for obtaining biologically safe and mechanically stable cell-laden structures.

To fabricate the spheroid-loaded cell constructs, we employed two processes: (1) printing of the BdECM-based bioink and (2) positioning the EC-laden mineral oil droplets on the valley consisting of two struts that were printed with the BdECM/TCP/hASC bioink. In this section, we demonstrate the cell viability and printing ability of the BdECM-based bioink obtained by controlling the pneumatic pressure of the barrel. To manipulate the pressure, we fixed the moving speed, barrel temperature, and working plate temperature at 5 mm/s, 25 °C, and 37 °C, respectively, as shown in Figure 3D. As shown in Figure 3E-F, after printing the hASCs/BdECM/β-TCP-bioink with various pneumatic pressures, cell viability on day 3 was determined for pneumatic pressures less than 120 kPa. The reason for the lower cell viability for relatively higher pneumatic pressures (over 120 kPa) was clearly the cell damage caused by the harsh wall-shear stress within the microsized nozzle (inner diameter = 250 μm) 35. Furthermore, in the bioprinting process, the printability of the bioink is considered a key criterion for successful printing. Figure 3G-H shows the printed struts for various printing pressures. As shown in the optical images, when the pressure was less than 90 kPa, stable printing struts were not formed. In addition, the diameter of the printed strut was linearly related to the printing pressure. Based on this result, we fixed the pneumatic pressure at 90 kPa, which is within the range of pressures at which a high cell viability is achieved, to fabricate a valley-type structure supporting the formation of cell spheroids.

**Figure 4:**
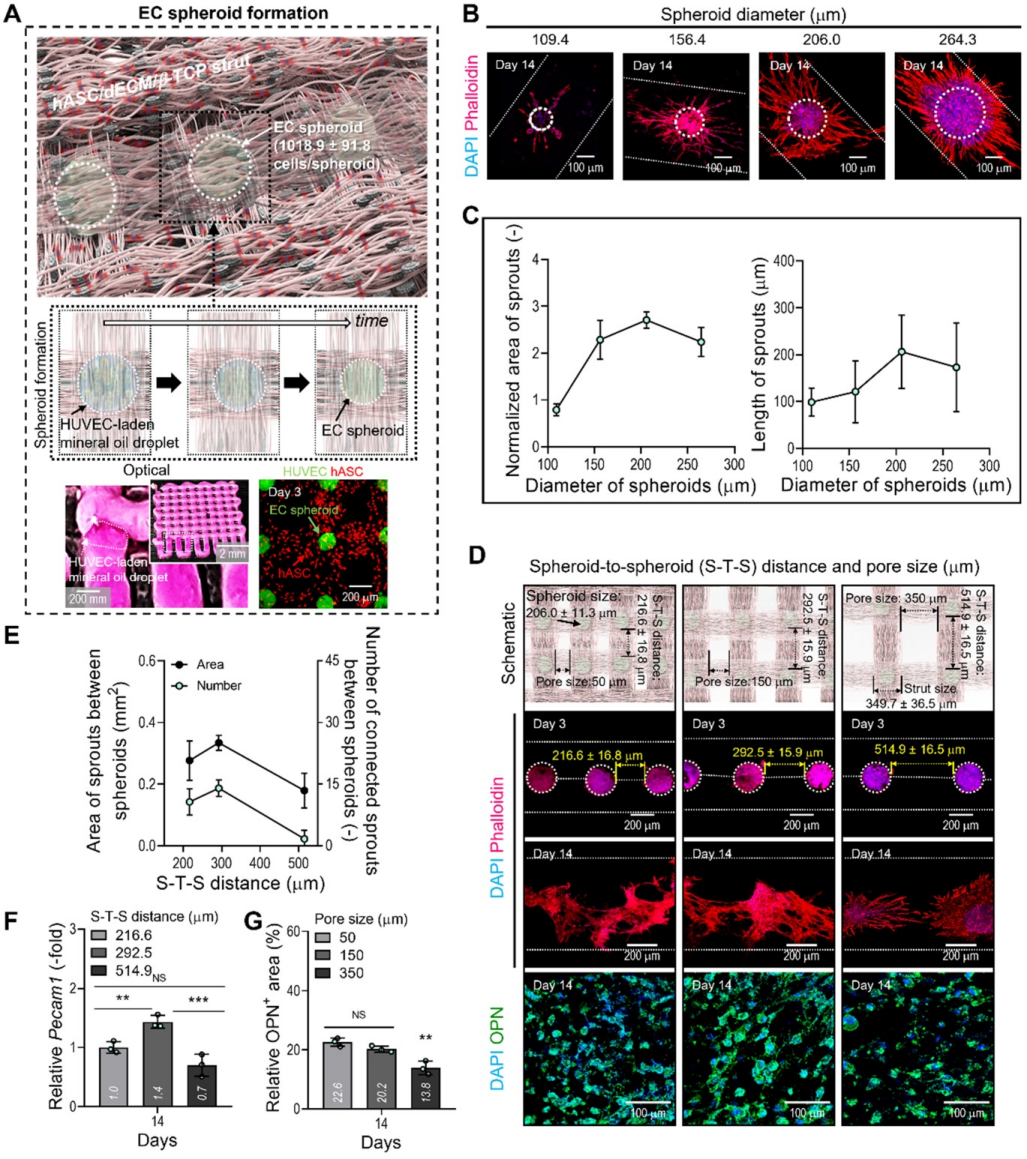
** Fabrication of a hybrid cell-structure using EC spheroids and hASCs. (A)** Schematics showing the formation of EC spheroids in the printed constructs. **(B)** Capillary sprout formation determined by dapi/phalloidin images for various diameters (109.6-264.4 µm) of the EC spheroids positioned on the bioprinted two-struts at 14 days. **(C)** Relative sprouting area normalized to the respective EC spheroid area (n = 10) and sprout length (n = 20). **(D)** The effect of various S-T-S distances (216.6, 292.5, and 514.9 µm) between EC spheroids on the sprout formation of ECs and OPN of hASCs. **(E)** Sprout area and connected sprout number between two spheroids (n = 3). **(F)** Relative *Pecam1* expression for each S-T-S distance at 14 days (n = 3). **(G)** Quantified OPN^+^ areas measured from the OPN images (n = 3). ^*^p < 0.05, ^**^p < 0.01, ^***^p < 0.001, one-way ANOVA with Tukey's HSD post-hoc test.

After fabricating the matrix structure consisting of two attached hASC-laden BdECM/TCP-struts, we deposited droplets of EC-laden mineral oil over the struts, as shown in the optical image in Figure 4A. To prepare the oil droplet, the nozzle moving speed and oil flow rate were 5 mm/s and 3.8 μL/min, respectively. After depositing the oil droplets on the struts, the BdECM/TCP/hASC struts were printed over the droplets of the cell-laden mineral oil. The image of CellTracker (HUVECs = green and hASCs = red) showed that the cell spheroids and hASCs were well composed in the printed construct shown in Figure 4A. This procedure was repeated several times to fabricate the 3D cell construct.

In general, the size of cell spheroids can affect the efficiency of angiogenesis; therefore, we evaluated the effect of spheroid size on capillary sprout formation. By controlling the volume flow rates of cell-laden mineral oil, we fabricated EC spheroids with different sizes, 109, 156, 206, and 264 μm, on the BdECM/β-TCP/hASC struts. After 14 days of cell culture, we measured the normalized sprout area divided by the spheroid size and sprout length (Figure 4B-C). As shown in the results, the spheroid size of 206 μm showed the most active sprouting of HUVECs. This phenomenon could be attained because the EC spheroids can provide the smallest hypoxic core region of various spheroid sizes. Similarly, the most suitable size of cell spheroids has been recommended as less than 200 μm in several studies 37, 38.

In addition, we observed the effect of distance between the EC spheroids (S-T-S distance) on capillary sprout formation and angiogenic gene expression because the spheroids (diameter = 206 μm; 1018.9 ± 91.8 cells/spheroid) could interact with each other in the BdECM/β-TCP/hASC struts. In that sense, the different distance between the EC spheroids laden in the structure can indicate the different pore size of the cell-construct, as shown in the schematic of Figure 4D. Figure 4D showed that the cell spheroids were observed at various S-T-S distances [216.6 ± 16.8 (pore size: 50 μm), 292.5 ± 15.9 (150 μm), and 514.9 ± 16.5 μm (350 μm)]. To assess capillary sprout formation at various distances between the cell spheroids, the sprout area and connected sprouts between the spheroids were measured. As shown in Figure 4E, at the S-T-S distance of 292.5 ± 15.9 μm, sprout formation was more active compared to that at other distances. In addition, the angiogenic gene, Pecam1, was also measured (Figure 4F). As expected, the same spheroid distance resulted in the highest gene expression level.

Generally, the number of EC spheroids can directly affect the osteogenic differentiation of the printed hASCs laden in the struts. To observe osteogenic activity at various pore sizes of the cell-constructs, the DAPI/OPN was measured at 14 days (Figure 4D-G). As shown in the results, the decreased OPN^+^ area of the cell-construct has been observed at the pore size of 350 μm (Figure 4G). The result could be occurred from the decreased number of EC spheroids in the cell-construct, resulting in the reduction of the amount of secreted signaling factors and the relatively poor cross-talks between the ECs and hASCs. From the result, we set the range of the S-T-S distance 300 ± 50 mm (pore size = 150 ± 50 mm) of the cell construct to induce endothelial and osteogenic differentiation effectively.

### *In vitro* cellular activities of hybrid cell-scaffolds

**Figure 5:**
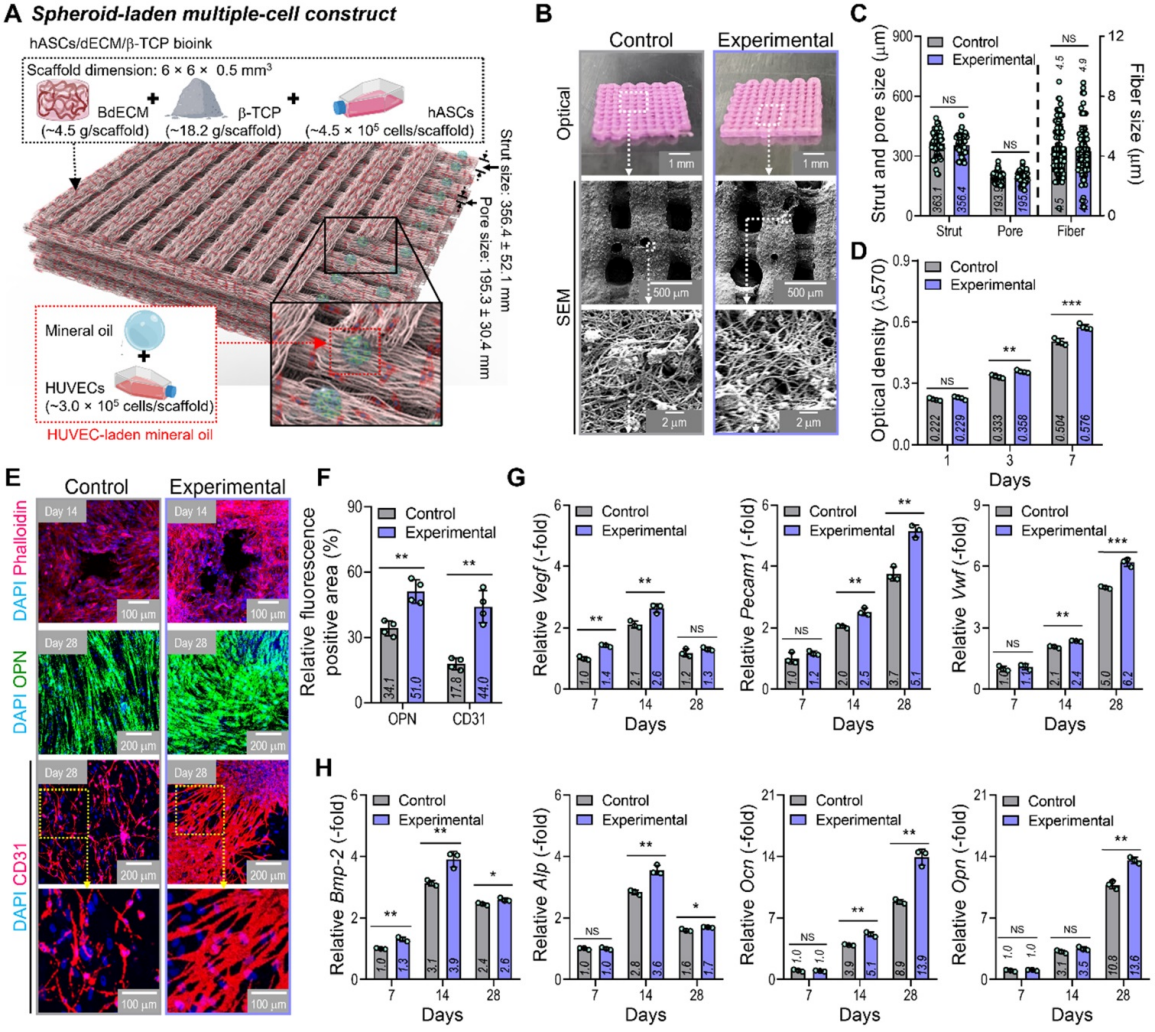
***In vitro* cellular activities of the cell-laden hybrid structure. (A)** Schematic exhibiting the designed EC spheroid-loaded cell construct (experimental construct). **(B)** Optical and SEM images of the control (multiple-cell-laden BdECM/β-TCP structure bioprinted using the mixture of hASCs and ECs) and experimental structure and **(C)** pore geometry (n = 50) and diameter of fibrillated fibers (n = 100) determined using the SEM images. **(D)** MTT assay of the structures (n = 4). **(E)** Dapi/phalloidin at 14 days, dapi/OPN at 28 days, and dapi/CD31 (red) at 28 days for the control and experimental groups. **(F)** Quantified OPN^+^ and CD31^+^ areas measured from the immunofluorescence images (n = 4). Expression level related to **(G)** angiogenic (*Vegf*, *Pecam1*, and *Vwf*) and osteogenic (*Alp*, *Bmp-2*, *Ocn*, and *Opn*) genes of the control and experimental groups at 7 and 14 days (n = 3). ^**^p < 0.01, ^***^p < 0.001, Student's *t*-test.

By using two cell-laden constructs in which a conventional bioprinted structure (control) had been fabricated using a bioink (BdECM/β-TCP/hASCS/ECs) and a hybrid cell construct (experimental) was fabricated using a bioink (BdECM/β-TCP/hASCs and EC spheroids; Figure 5A), several cellular activities, including cell proliferation, angiogenic factors, and osteogenic activities, were compared. Figure 5B shows the optical/SEM images of the fabricated cell constructs, and the microfibrous morphological structure obtained due to the fibrillated collagen component of the dECM. The pore geometry (pore and strut size) of the fabricated structures was similar for those of the control and experimental groups, as shown in Figure 5C. For the cell constructs, cell proliferation was determined using an MTT assay, and significantly higher proliferation was assessed in the experimental group with cell spheroids than the control (Figure 5D). DAPI (blue)/phalloidin (red), DAPI/OPN (green), and DAPI/CD31 (red) immunofluorescence images of the cell constructs after several culture periods are shown in Figure 5E. The immunofluorescence images showed that F-actin was more dynamically formed in the experimental group than in the control group. Additionally, the positive areas of OPN and CD31 were significantly greater in the experimental structure than in the control (Figure 5F). Figure 5G-H shows the expression levels of angiogenic (*Vegf*, *Pecam1*, and *Vwf*) and osteogenic (*Alp*, *Bmp-2*, *Ocn*, and *Opn*) genes. The experimental group with the EC spheroids showed significantly higher gene expression levels than the control.

### *In vivo* bone formation in a mastoid obliterated rat model

**Figure 6:**
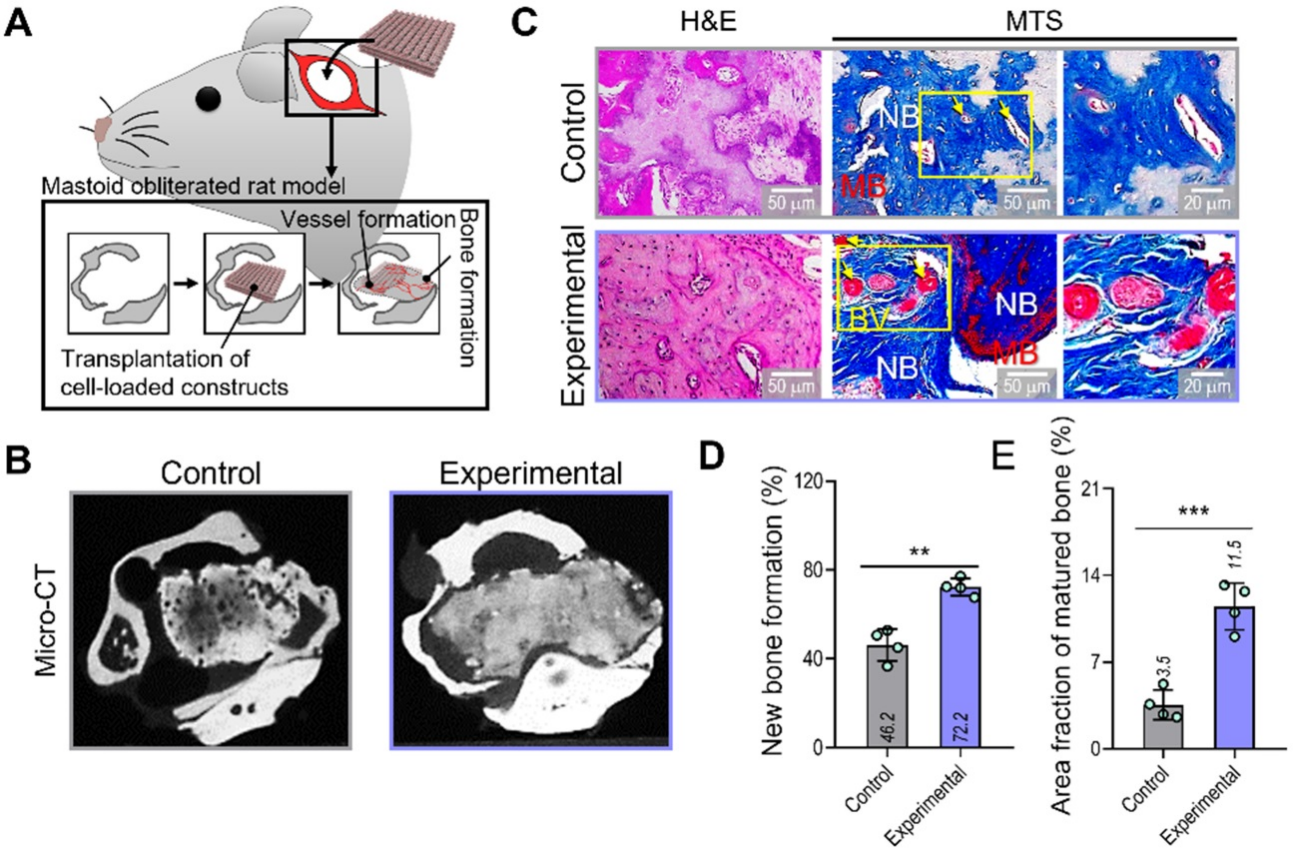
**Bone and vessel formation in the hybrid cell-loaded biocomposites in a mastoid obliterated rat model. (A)** Schematics illustrating the transplantation of the control and experimental biocomposites into a mastoid obliterated rat model. **(B)** Microcomputed tomography images of the transplanted regions at 8 weeks. **(C)** Histological staining images (H&E and MTS) of the implanted constructs at 8 weeks. Yellow arrows indicate developed vessels, and “NB,” “BV,” and “MB” indicate new bone, blood vessel, and matured bone, respectively. **(D)** Areas of newly formed and **(E)** maturated bone measured from MTS images (n = 4). ^**^p < 0.01, ^***^p < 0.001, Student's *t*-test.

To evaluate the therapeutic effects of the spheroid-based cell construct on *in-vivo* bone formation, we implanted two cell-loaded constructs (control and experimental) in a rat mastoid-obliterated model (Figure 6A). Eight weeks after implantation, new bone formation was evaluated by observing micro-CT images (Figure 6B). Newly developed bone structures were observed in the transplanted region of the bulla cavity for both types of cell-loaded constructs, and bone formation was highly activated in the experimental structure. To observe bone and vessel formation more precisely, histological assessments were performed using H&E and MTS (Figure 6C). In both groups, the implanted bone constructs were significantly degraded at 8 weeks. Significantly more newly developed bone (72.2%) and a larger area of mature bone (11.5%) were observed in the experimental than in the control group (new bone formation,46.2%; mature bone area,3.5%) (Figure 6D,E).

**Figure 7:**
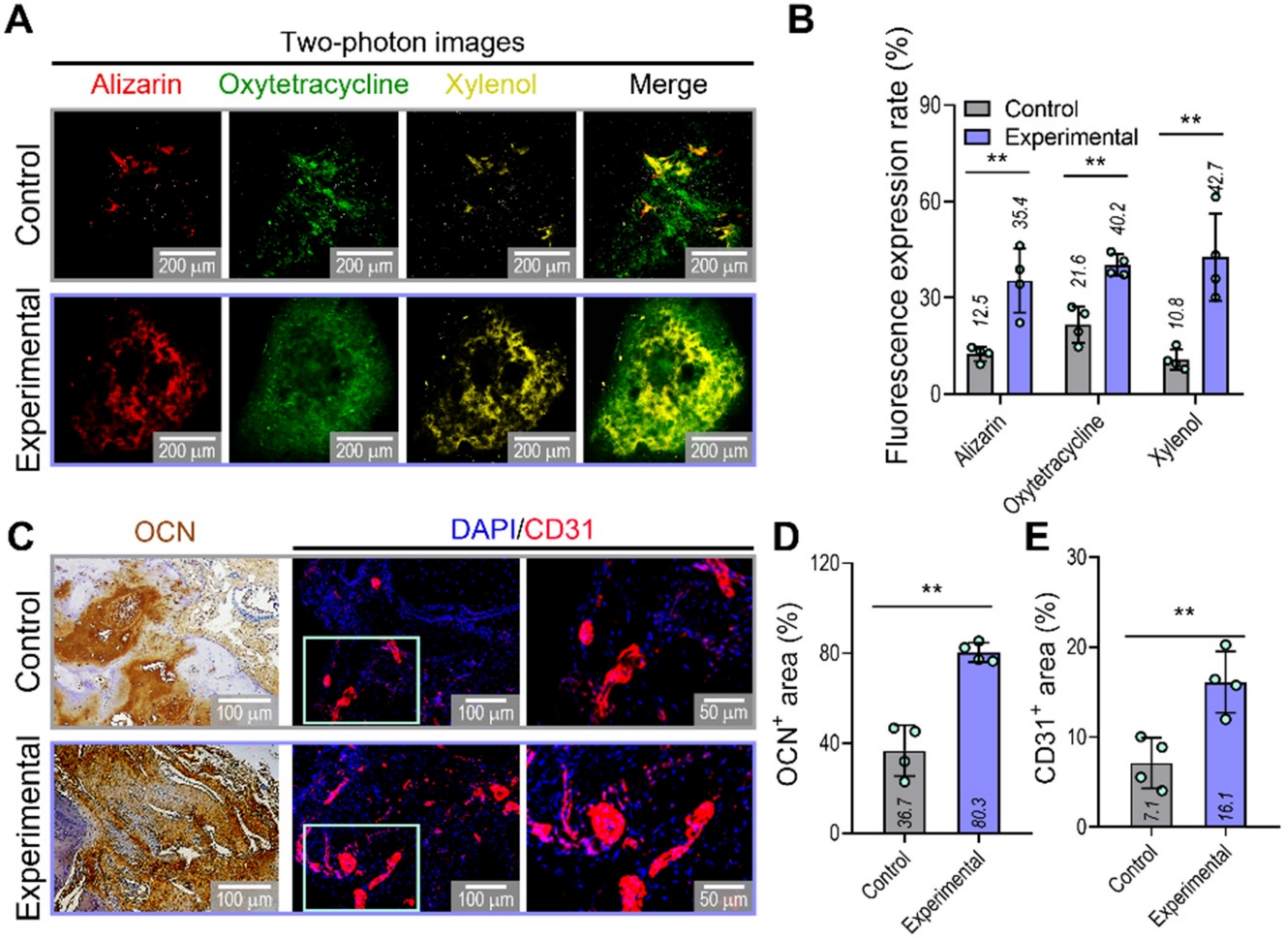
**Visualization of developed bone and vessels in the mastoid obliterated rat model. (A)** Alizarin (red)-, oxytetracycline (green)-, and xylenol (yellow)-stained two-photon fluorescence images and **(B)** quantitative analysis of stained areas indicating bone deposition in transplanted constructs (n = 4). **(C)** Immunohistochemical (OCN; biotin) and immunofluorescence (CD31; red) images of constructs in the transplanted groups at 8 weeks. Quantification of **(D)** OCN-positive and **(E)** CD31-positive areas measured from the OCN and CD31 images (n = 4). ^**^p < 0.01, Student's *t*-test.

To confirm the ability of implanted prevascularized bone constructs to induce endochondral ossification and angiogenesis, we performed alizarin (red), oxytetracycline (green), xylenol (yellow), osteocalcin (OCN), and DAPI/CD31 staining (Figure 7A-E). Although newly developed and calcified bone was detected by two-photon imaging in both groups, greater bone deposition, marked by alizarin, oxytetracycline, and xylenol, was observed in the experimental (alizarin,35.4%; oxytetracycline,40.2%; xylenol,42.7%) than in the control group (alizarin,12.5%; oxytetracycline,21.6%; xylenol,10.8%) (Figure 7B). Similar to the histological results, the experimental construct exhibited significantly higher expression of OCN and CD31, as shown by the quantification of the labeled areas in the images for OCN^+^ (control:36.7%, experimental:80.3%) and CD31^+^ (control:7.1%, experimental:16.1%) (Figure 7D,E). The results suggested that the experimental construct with EC spheroids exhibited improved secretion of angiogenic- and osteogenic-related paracrine factors evaluated in the *in-vitro* analyses and that they were more effective in promoting the development of new bone and vessels than the control construct fabricated using bioink containing a simple mixture of HUVECs and hASCs.

Based on the results of *in vitro* and *in vivo* vessel and bone formation, we can suggest that the spheroid-laden multiple cell constructs can be applied to bone tissue remodeling. In addition, the applications of the proposed hybrid cell constructs can be expanded to regenerative theranostics *via* further modifications. Cooperation of the cell-loaded bioinks with additional nanoparticles, cellular tracking techniques, or imaging agents for bioceramics and proteins in the constructs will enable the personalized platform of monitoring and treating diseases in the future 39-41.

## Conclusion

We successfully developed hybrid cell constructs consisting of EC spheroids and homogeneously distributed hASC-laden dECM/β-TCP struts to obtain effectively vascularized bone tissues. The geometrical size of the EC spheroids and the distance between the spheroids laden in the cell struts were appropriately selected in terms of capillary sprout formation and angiogenic gene expression. The newly designed cell constructs supplemented with EC spheroids and hASCs clearly enhanced bone formation with significant angiogenic activities compared to conventionally bioprinted cell constructs fabricated using a mixture of hASCs and ECs. Based on the results presented here, the hybrid cell constructs supporting the hASC/EC spheroid co-culture can be a potential therapeutic biomaterial for effectively inducing vascularized bone tissues.
