# Supplementary material for: Bone tissue engineering supported by bioprinted cell constructs with endothelial cell spheroids
Source: Theranostics. 2022 Jul 11;12(12):5404–17. doi: 10.7150/thno.74852 (PMC9330514; doi:10.7150/thno.74852)
Supplement: Supplementary file 1 — Supplementary figure and table. [file thnov12p5404s1.pdf]

Supplementary materials

Bone tissue engineering supported by bioprinted cell constructs with endothelial cell spheroids

WonJin Kim,[a,1] Chul Ho Jang,[b,1] GeunHyung Kim[a,c,*]

[a]Department of Biomechatronic Engineering, College of Biotechnology and Bioengineering, Sungkyunkwan University (SKKU), Suwon 16419, South Korea

[b]Department of Otolaryngology, Chonnam National University Medical School, Gwangju 61469, South Korea

[c]Biomedical Institute for Convergence at SKKU (BICS), Sungkyunkwan University, Suwon 16419, South Korea

[*]Corresponding author at: Department of Biomechatronic Engineering, College of Biotechnology and Bioengineering, Sungkyunkwan University (SKKU), Suwon 16419, South Korea. E-mail address: gkimbme@skku.edu (G. Kim)

[1]The authors contributed equally to this work.

Materials and methods

Preparation of BdECM

Before preparing bone-specific bioinks, bone tissues were isolated from the lower limbs of Yorkshire pigs (female, 10–15 months old) and crushed to obtain bone powder after rinsing several times with Dulbecco's phosphate-buffered saline (DPBS; Biowest, USA) and deionized water (DW). The bone powder was demineralized by treatment with 0.5 M HCl (Sigma-Aldrich, USA) for 5 h with continuous stirring at 27 °C. Following the removal of the remaining solution, the treated powder was washed at least three times with DW. The remaining lipid in the powder was removed by treatment with a lipid removal solution containing chloroform (Sigma-Aldrich) and methanol (Sigma-Aldrich) at a ratio of 1:1. After 1 h of treatment, the solution was rinsed with methanol five times with DW, and then freeze-dried in a freeze-drier (SFDSM06; Samwon, South Korea). The demineralized bone matrix (DBM) was stored at -80 °C before decellularization.

Lyophilized DBM was incubated in trypsin (0.05%)-ethylenediamine tetra-acetic acid (0.02%; EDTA) (TE; Sigma-Aldrich) solution for 2 h at 37 °C. After removing the TE solution, the DBM powder was washed thrice with DW and soaked in 70% ethanol for 1 day. The powder was then washed three times with DW, lyophilized, and stored at -80 °C before solubilization. The freeze-dried decellularized tissue was digested in pepsin solution (0.1% w/v in 0.5 M acetic acid; Sigma-Aldrich) at room temperature for 2 days, and precipitation was performed by adding sodium chloride (Sigma-Aldrich). The solution was dialyzed (1000 kDa molecular cut-off; Spectrum Chemical Manufacturing, USA) at 4 °C for 3 days. The dialyzed soluble bone dECM was lyophilized and stored at -80 °C.

To observe the osteogenesis of hASCs, cells (1.2×10^7 cells/mL) were loaded into collagen (5 wt%) and BdECM (5 wt%) hydrogels. The hASC-loaded bioinks were cultured with GM at 37 °C under 5% CO_2, and the medium was changed every 2 days.

Tables

Table S1. Primer sequences.

Gene	Source	Primer sequence		GeneBank number
		Left (5′ – 3′)	Right (5′ – 3′)	
Gapdh	*Homo sapiens*	CCATGGGGAAGGTGAAGGTC	AGTGATGGCATGGACTGT	NM_002046.7
Pecam1	*Homo sapiens*	TGAGTGGTGGGCTCAGATTG	TGAGTCTAGGTCGGGGAGTG	NM_000442.5
Vegf	*Homo sapiens*	AGGCCAGCACATAGGAGAGA	ACGCGAGTCTGTGTTTTTGC	NM_001171623.1
Vwf	*Homo sapiens*	ACACCTGCATTTGCCGAAAC	ATGCGGAGGTCACCTTTCAG	NM_000552.5
Opn	*Homo sapiens*	AAGTTTCGCAGACCTGACATC	GGGCTGTCCCAATCAGAAGG	NM_000582.2
Alp	*Homo sapiens*	GGCACCTGCCTTACTAACTCC	CTTGCCACGTTGGTGTTGA	NM_000478.6
Bmp-2	*Homo sapiens*	CAGACCACCGGTTGGAGA	CCACTCGTTTCTGGTAGTTCTTC	NM_001200.4
Ocn	*Homo sapiens*	TGAGAGCCCTCACACTCCTC	ACCTTTGCTGGACTCTGCAC	NM_199173.6

Gapdh: glyceraldehyde-3-phosphate dehydrogenase; *Pecam1*: platelet and endothelial cell adhesion molecule 1 (CD31); *Vegf*: vascular endothelial growth factor; *Bmp-2*: bone-morphogenic protein 2; *Cxcl12*: C-X-C motif chemokine ligand 12 (SDF-1); *Vwf*: von Willebrand factor; *Opn*: bone sialoprotein I (*Spp1*); *Alp*: alkaline phosphatase; *BMP-2*: bone morphogenic protein 2; *Ocn*: bone gamma-carboxyglutamate protein (*Bglap*).

Figures

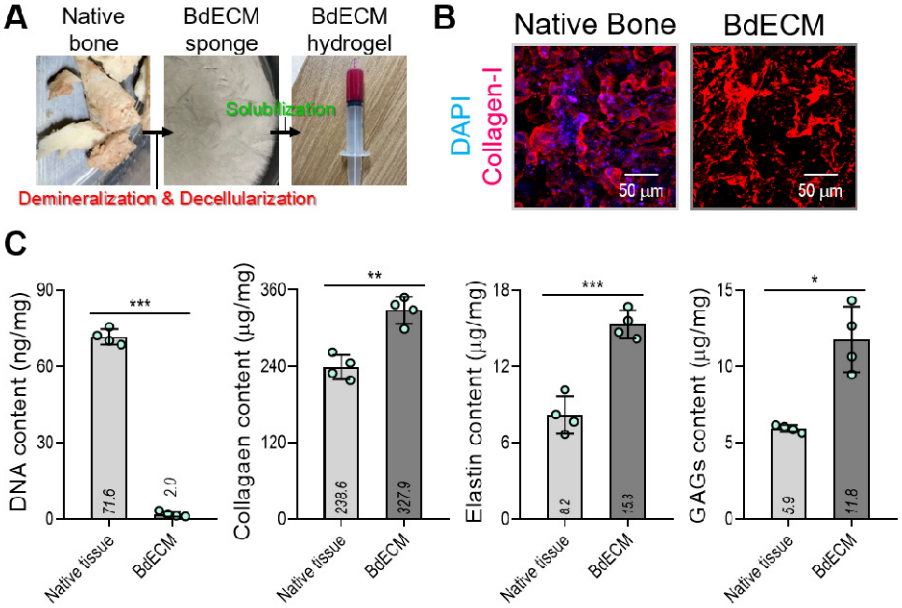

Figure S1. Characterization of BdECM bioink. (A) Optical images demonstrating the preparation of BdECM from porcine leg bone tissue *via* demineralization, decellularization, and solubilization processes. (B) Dapi/collagen-I (red) images of native bone and BdECM. (C) Cellular (DNA) and ECM (collagen, elastin, and glycosaminoglycans) contents of BdECM (n = 4). $^{*}p < 0.050$, $^{**}p < 0.010$, $^{***}p < 0.001$, Student's *t*-test.
